# *Rhodococcus erythropolis* Encephalitis in Patient Receiving Rituximab

**DOI:** 10.3201/eid1808.110434

**Published:** 2012-08

**Authors:** Satish R. Bagdure, Mark A. Fisher, Michael E. Ryan, Faisal A. Khasawneh

**Affiliations:** Texas Tech University Health Sciences Center, Amarillo, Texas, USA (S.R. Bagdure, M.E. Ryan, F.A. Khasawneh);; University of Utah, Salt Lake City, Utah, USA (M.A. Fisher);; and Associated Regional and University Pathologists Laboratories, Salt Lake City (M.A. Fisher)

**Keywords:** Rhodococcus erythropolis, encephalitis, meningoencephalitis, rituximab, bacteria, rheumatoid arthritis

**To the Editor:** *Rhodococcus* spp. infections occur predominantly in immunocompromised patients, and most infections are caused by *Rhodococcus equi* (*1*). Seven cases of *R. erythropolis* infections in humans have been described (*2**–**8*). None of these cases included a central nervous system infection (Table). We report a case of *R. erythropolis* meningoencephalitis in a patient with rheumatoid arthritis who was treated with rituximab and methotrexate.

**Table:** Characteristics of 7 patients infected with *Rhodococcus erythropolis*

Patient no.	Age, y/sex	Cconcurrent condition	Characteristic	Reference
1	63/M		Peritoneal dialysis, catheter-related peritonitis	(*2*)
2	44/F		Exacerbation of bronchiectasis	(*3*)
3	24/M	HIV/AIDS	Disseminated skin infection	(*4*)
4	82/F		Chronic endophthalmitis after lens implantation	(*5*)
5	53/F		Osteomyelitis after first metatarsophalangeal joint arthrodesis	(*6*)
6	6/M	Acute lymphocytic leukemia	Catheter-related bloodstream infection	(*7*)
7	79/M	Esophageal cancer	Catheter-related bloodstream infection	(*8*)

In September 2010, a 44-year-old woman in Amarillo, Texas, USA, with rheumatoid arthritis who was being treated with rituximab and methothrexate was hospitalized with a 5-day history of fever, headache, and confusion. Physical examination showed that the patient was febrile (38.3°C) and drowsy. Results of a computed tomography scan of the brain were normal. Cerebrospinal fluid (CSF) obtained by lumbar puncture showed a leukocyte count of 112 cells/mm^3^ (28% neutrophils, 56% lymphocytes) and glucose and total protein concentrations of 64 mg/dL and 81 mg/dL, respectively.

At admission, the patient was given a diagnosis of meningoencephalitis and treated with vancomycin, meropenem, ampicillin, and acyclovir. Magnetic resonance imaging of the brain showed diffuse cortical increased signal on T2‐weighted imaging and confluent, near-symmetric T2 signal hyperintensities in the thalami extending into the brain stem that demonstrated mild post contrast enhancement and no evidence of restricted diffusion. This finding was believed to be suggestive of viral encephalitis.

The mental status of the patient deteriorated and she became profoundly weak but had preserved reflexes. She had no movement in her lower extremities and only limited movements in her upper extremities. She was transferred to the intensive care unit for intubation and mechanical ventilation.

Routine CSF bacterial and viral cultures were negative. Serologic results for cryptococcal antigen and West Nile virus in CSF were negative. PCR results for herpes simplex virus, cytomegalovirus, and enteroviruses in CSF were negative.

The patient became afebrile and showed slow but limited improvement. She became more alert and occasionally raised her right index finger on command. After CSF bacterial cultures were reported negative, antimicrobial drugs were discontinued. Five days later, while the patient was still receiving mechanical ventilation, fever relapsed, prompting treatment and tests for a hospital-acquired infection.

A repeat lumbar puncture was conducted 10 days after admission. Opening pressure was 340 mm H_2_O. The leukocyte count was 167 cells/mm^3^ (2% monocytes, 98% lymphocytes), and glucose and total protein concentrations were 51 mg/dL and 103 mg/dL, respectively. CSF from the repeat lumbar puncture was used for routine bacterial culture but not mycobacterial culture. There were no obvious infection foci and antimicrobial drugs were discontinued.

On the 18th day of hospitalization, the patient became unresponsive and had fixed dilated pupils. Computed tomography scan of the brain showed obstructive hydrocephalus and cerebellar herniation. The patient died 1 day later. During the third week of incubation, gram-positive rods grew in mycobacterial broth medium and were subsequently identified as *R. erythropolis*.

Species of the genus *Rhodococcus* (order Actinomycetales, family *Nocardiaceae*) are aerobic, gram-positive, partially acid-fast, coccoid to rod-shaped bacteria (*9*). They have been isolated from a variety of sources (*6*). *Rhodococcus* spp. are generally considered to have low virulence (*7*). Most documented human infections with *Rhodococcus* spp. have been caused by *R. equi*, and pneumonia is the most commonly described condition (*7*).

*R. erythropolis* is typically found in soil and has been detected on the surface of the healthy human eye, but there are no reports of its presence at other sites in humans (*7*). *R. erythropolis* colonies are typically rough and orange to red; thus, the name *erythropolis*, which means red city (*8*).

Immunosuppression is a major risk factor in the pathogenesis of *Rhodococcus* spp. infections (*10*). The patient had been treated with rituximab and methotrexate for 2 years. Multiple infectious complications have been described for each of these drugs, but only 1 case of *Rhodococcus* infection has been reported for a patient receiving methotrexate and none have been described for patients receiving rituximab (*10*).

Because of difficulties in species identification and delays in growth, non–*R. equi* infections might be underdiagnosed (*9*). This finding is complicated by the fact that these gram-positive bacilli may be misidentified as contaminating diphtheroids (*9*). It is unlikely that this organism was a contaminant, given that the fever in the patient relapsed after antimicrobial drugs were discontinued and no other cause was identified. The isolate from the patient was identified by sequencing the first ≈500 bp of the 16S rRNA gene, which is a useful molecular technique for speciation of the genus *Rhodococcus* (*9*). The isolate showed 99.8% identity with *R. erythropolis* type strain DSM 43066.

Antimicrobial drug susceptibility patterns in non–*R. equi* rhodococci have not been studied, and there are no established standards for treating patients with *Rhodococcus* spp. infections. This case, along with previously reported cases, represents emergence of an opportunistic pathogen in a rapidly increasing patient population, namely those with impaired local or systemic immunity.
